# Potential Role of Seven Proteomics Tissue Biomarkers for Diagnosis and Prognosis of Prostate Cancer in Urine

**DOI:** 10.3390/diagnostics12123184

**Published:** 2022-12-16

**Authors:** Ivo Vujicic, Aleksandar Rusevski, Oliver Stankov, Zivko Popov, Aleksandar Dimovski, Katarina Davalieva

**Affiliations:** 1 University Clinic for Urology, University Clinical Centre “Mother Theresa”, 1000 Skopje, North Macedonia; 2 Research Centre for Genetic Engineering and Biotechnology “Georgi D Efremov”, Macedonian Academy of Sciences and Arts, 1000 Skopje, North Macedonia; 3 Clinical Hospital “Acibadem Sistina”, 1000 Skopje, North Macedonia; 4 Medical Faculty, University “St. Cyril and Methodius”, 1000 Skopje, North Macedonia; 5 Macedonian Academy of Sciences and Arts, 1000 Skopje, North Macedonia; 6 Faculty of Pharmacy, University “St. Cyril and Methodius”, 1000 Skopje, North Macedonia

**Keywords:** prostate cancer, proteomics, biomarker, non-invasive diagnosis, non-invasive prognosis, ELISA

## Abstract

As the currently available tests for the clinical management of prostate cancer (PCa) are still far from providing precise diagnosis and risk stratification, the identification of new molecular marker(s) remains a pertinent clinical need. Candidate PCa biomarkers from the published proteomic comparative studies of prostate tissue (2002–2020) were collected and systematically evaluated. AZGP1, MDH2, FABP5, ENO1, GSTP1, GSTM2, and EZR were chosen for further evaluation in the urine of 85 PCa patients and controls using ELISA. Statistically significant differences in protein levels between PCa and BPH showed FABP5 (*p* = 0.019) and ENO1 (*p* = 0.015). A biomarker panel based on the combination of FABP5, ENO1, and PSA provided the highest accuracy (AUC = 0.795) for PCa detection. The combination of FABP5, EZR, AZGP1, and MDH2 showed AUC = 0.889 in PCa prognosis, with 85.29% of the samples correctly classified into low and high Gleason score (GS) groups. The addition of PSA to the panel slightly increased the AUC to 0.914. AZGP1, FABP5, and EZR showed significant correlation with GS, stage, and percentage of positive biopsy cores. Although validation using larger patient cohorts will be necessary to establish the credibility of the proposed biomarker panels in a clinical context, this study opens a way for the further testing of more high-quality proteomics biomarkers, which could ultimately add value to the clinical management of PCa.

## 1. Introduction

Prostate cancer (PCa) is defined as the second most commonly diagnosed cancer and the fifth leading cause of mortality in male population worldwide [1]. The current challenges in the clinical management of this disease are due to the limited specificity of the prostate-specific antigen (PSA) for detecting PCa. This lack of specificity contributes to the over- or under-treatment associated with the medical costs and psychological distress of patients [2]. Although PSA testing has greatly increased the number of men diagnosed with PCa [3], among diagnosed cancer cases, around 45% present with an indolent course, which is unlikely to progress in the absence of curative treatment [4]. On the other hand, 15% of PCa cases occur in men with normal serum PSA levels [5]. Efforts to improve PCa detection and prognosis resulted in extensive research that, so far, has led to the development of several tests that have become commercially available in recent years (extensively reviewed in [6]). Although progress has been made, most of these tests have not been fully integrated into clinical practice and further prospective studies are recommended to validate their clinical utility and cost-effectiveness [7]. Moreover, half of these tests are biopsy-based and, therefore, have shortcomings associated with invasive sampling and variable performance, due to the tissue heterogeneity. On the other hand, most of the non-invasive body fluid-based tests are centered on measurements of PSA isoforms [6]. Although PSA isoforms appear to be of use, they are still more ‘prostate specific’ than ‘cancer specific’ and are not useful in the stratification of PCa risk [8]. In addition to the above, the currently available tests are only applicable in a repeat biopsy setting.

Therefore, the identification of molecular marker(s) that can function as a non-invasive clinical tool to aid in the management of PCa diagnosis and treatment remains a crucial clinical need. It has been widely accepted now that understanding the pathophysiology of PCa as a complex, heterogenic disease and the discovery of more sensitive/specific tools for disease detection require a systemic approach. Comparative proteomics studies have a significant role in this by aiming to detect and quantify proteins with altered abundance without prior biological knowledge, which subsequently may reveal credible candidate biomarkers. In this context, comparative analysis of samples from well-defined groups of patients with PCa, using high-throughput proteomics technologies, has shown great potential for the discovery of new, non-invasive testing. Proteomics approaches have been widely employed in PC biomarker research, as reflected by the numerous studies that have been published over the last 20 years (extensively reviewed in [9,10]). The research has focused on the development of urinary, blood, seminal plasma, and tissue biomarkers, in the context of the diagnosis, risk stratification, and prediction of treatment response. As a result of these investigations, a vast amount of proteomics data has been collected, and some of the shortlisted biomarker candidates were verified in independent cohorts. The initial assessment of their clinical utility showed rather good performance [10]. 

Since large amounts of proteomics data have been generated for PCa and multiple proteins have been defined as potential biomarkers, the next logical step should be to move towards the testing of these markers in the specific clinical context in well-designed, appropriately powered prospective studies. It is also becoming evident from multiple studies, that the combination of multiple markers into a panel increases the performance of the tests. This study aimed to collect the candidate PCa biomarkers from the published proteomic comparative studies analyzing prostate tissue and preselect the most promising candidates for PCa detection and prognosis based on: (1) prostate tissue specificity, (2) identification as differentially abundant in PCa in at least two or more independent studies analyzing prostate tissue; (3) and detection into body fluids, particularly in urine. The pre-selected candidates were measured in the urine of well-selected groups of PCa patients and controls, in order to assess their sensitivity and specificity for detecting PCa, as well as to give the prognosis, in regard to the progression of the disease towards the invasive forms.

## 2. Materials and Methods

### 2.1. Study Design and Selection of Candidates

A systematic literature search was performed using the PubMed database in January 2020. Manuscripts were retrieved based on the search terms (prostate cancer) OR (prostate adenocarcinoma) AND (proteomics) AND (tissue) AND (biomarker). The retrieved articles were prescreened to include the following criteria: (1) discovery proteomics studies and (2) human prostate specimens. The exclusion criteria included: (1) animal studies and (2) studies performed on cell cultures (treated or untreated). The selected studies were screened for the differentially abundant proteins in PCa group(s), compared with controls, and these were extracted together with their regulation trend and data from validation in an independent cohort, if available. Tissue biomarkers were aligned according to the number of identifications by independent studies and further preselected to include only the proteins with three or more identifications. Next, the prostate tissue specificity and subcellular localization of the candidate biomarkers were evaluated based on protein localization of the corresponding proteins in normal and tumor prostate tissue using the publicly available database The Human Protein Atlas ver. 19 [11]. Tissue specificity, subcellular localization, molecular functions, and biological processes of the candidate biomarkers were also overviewed using the UniProt Knowledgebase (UniProtKB). In addition, protein detection in urine was evaluated according to the available data from the Human Protein Reference Database [12]. The candidates for ELISA testing in urine were selected based on their number of identifications by independent studies, uniformity of the observed regulation trend in PCa, protein detection in urine, and inclusion in some of the commercial tissue-based gene assays for PCa detection and stratification, such as ConfirmMDX [13], OncotypeDX [14], or Prolaris [15].

### 2.2. Patient Data and Samples

The urine samples were collected from patients with clinically and histologically confirmed PCa and BPH obtained from the University Clinic for Urology, University Clinical Centre “Mother Theresa”, Skopje, North Macedonia, in the period from June 2019 until December 2020. The diagnosis of the patients was based on the histological evaluation of the tissues obtained by biopsy or surgical procedure. Patients’ clinical records, including age, diagnosis, number of positive biopsy cores (in regard to total collected cores), histology grading, Gleason score (GS), and pre-biopsy serum PSA, were reviewed to preselect the urine samples used in this study (Supplementary Table S1). The control group consisted of BPH patients, preselected to be without signs of inflammation (prostatitis). The samples from PCa patients were divided into four groups, according to the Gleason score, starting from GS6 to GS9. 

The urine samples (3–10) mL were collected from the patients prior to prostate biopsies and stored on ice for short period (<1 h). Samples were centrifuged at 1000× *g* for 10 min to remove cell debris, aliquoted in 1.5 mL tubes, and stored at −80 °C until use.

### 2.3. Quantitative Measurement of Candidate Proteins in Urine

For the quantitative measurement of the selected proteins in urine, we used the following ELISA kits: Alpha-2-Glycoprotein 1, Zinc Binding (AZGP1) human ELISA Kit from CLOUD-CLONE CORP (SEL231Hu) with limit of detection (LOD) = 1.8 ng/mL; Malate Dehydrogenase 2 (MDH2) human ELISA Kit from CLOUD-CLONE CORP (SEH672Hu) with limit of detection (LOD) = 0.28 ng/mL; Fatty Acid Binding Protein 5, Epidermal (FABP5) human ELISA Kit from CLOUD-CLONE CORP (SEB985Hu) with limit of detection (LOD) = 0.117 ng/mL; Enolase 1 (ENO1) human ELISA Kit from Antibodies-online GmbH (ABIN6574277) with limit of detection (LOD) = 0.31 ng/mL; Glutathione S Transferase Pi (GSTP1) human ELISA Kit from Antibodies-online GmbH (ABIN6574253) with limit of detection (LOD) = 0.15 ng/mL; Cytovillin/ezrin (EZR) human ELISA Kit from CUSABIO TECHNOLOGY LLC (CSB-E09922h) with limit of detection (LOD) = 0.078 ng/mL; Glutathione S-transferase Mu 2 (GSTM2) human ELISA Kit from CUSABIO TECHNOLOGY LLC (CSB-EL009981HU) with limit of detection (LOD) = 0.078 ng/mL. Samples were assayed using 100 µL undiluted urine, in duplicate, according to the manufacturer’s instructions. The concentrations of AZGP1, MDH2, FABP5, ENO1, GSTP1, EZR, and GSTM2 were normalized to urine creatinine to correct for variations in urinary concentration. The level of creatinine in urine was determined using the Jaffé method by Crea Jaffe Gen. 2 Urine Kit (Roche Diagnostics) with LOD = 0.01 mmol/L.

### 2.4. Statistical Analysis

Statistical analyses were performed using XLSTAT software ver. 2022.1.2 [16]. The distribution of the data for each tested protein was tested using Shapiro–Wilk, Anderson–Darling, Lilliefors, and Jarque–Bera tests. To determine if there were statistically significant differences at the level of tested proteins between tested groups, nonparametric Kruskal–Wallis test was performed using Dunn’s test for the multiple comparisons and Bonferroni correction. Two-sample comparisons were performed using Mann–Whitney U-test. To estimate the combined diagnostic potential of candidate biomarkers, logistic regression analyses were performed with clinical diagnosis or Gleason grading as the dependent variable and protein concentrations in urine as independent variables. Receiver operating characteristic (ROC) curves were used to define the diagnostic performance given by areas under the curves (AUC). A confidence level of 95% (*p* < 0.05) was considered significant for all performed tests. The Spearman correlation was used to assess the association between levels of the tested proteins in urine, as well as with clinical parameters such as age, serum PSA, GS, stage of the disease, and percentage of positive biopsy cores. 

To test if the study was sufficiently powered, in terms of sample size, we have performed post-hoc power analyses using free software G*Power ver. 3.1.9.7. [17] with the following inputs: statistical test means: Wilcoxon–Mann–Whitney test (two groups); tails: one; parent distribution: Laplace; α = 0.05; effect size (d) calculated from means and standard deviation of the two groups; sample sizes of the two compared groups.

## 3. Results

### 3.1. Meta-Analysis of PCa Tissue Proteomics Studies

**Figure 1:**
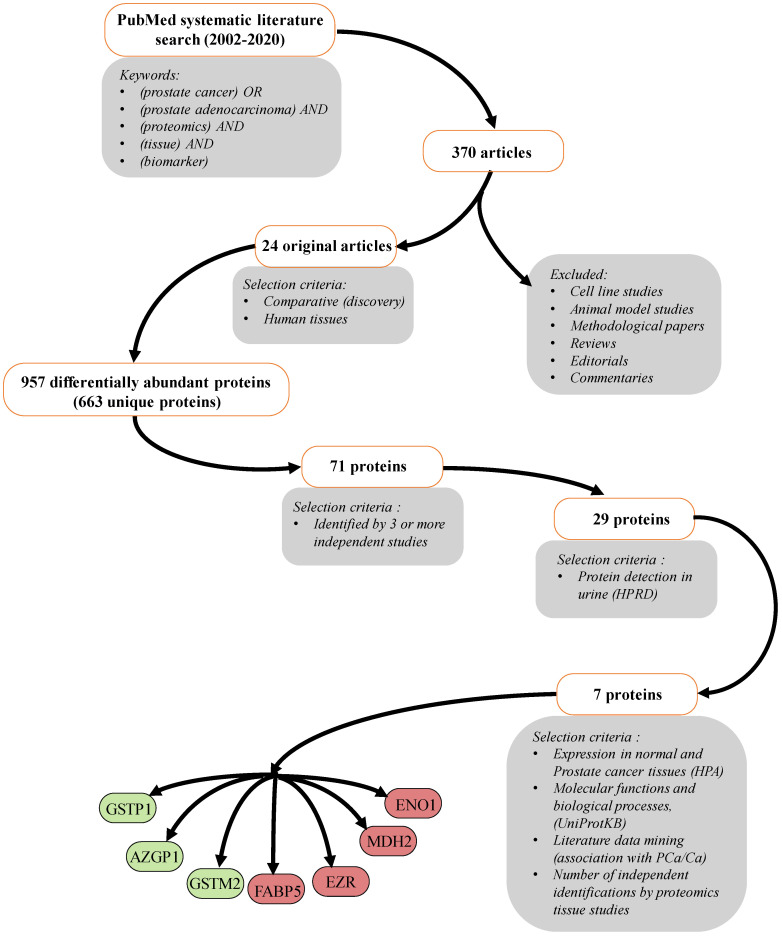
Study design and workflow of the meta-analysis. The color of the selected proteins denotes the observed regulation trend (red—up-regulated, green—down-regulated) in PCa by proteomics studies.

To obtain an unbiased, comprehensive, and thorough overview of the identified proteomics biomarkers for PCa detection and progression, we have performed a systematic literature search using the PubMed database. The study design and workflow are represented in Figure 1. The systematic literature search retrieved a total of 370 articles. These were further manually screened to include only comparative (discovery) proteomics studies performed on human tissues. Methodological papers, reviews, editorials, commentaries, and studies performed only in cell lines/animal models were excluded, leading to the list of 24 original articles published in a timespan from 2002–2018 [18,19,20,21,22,23,24,25,26,27,28,29,30,31,32,33,34,35,36,37,38,39,40,41].

We extracted a total of 957 differentially abundant proteins from these studies, which corresponded to 663 unique proteins. Differentially abundant proteins were sorted according to the number of independent identifications, together with the observed regulation trend in PCa (Supplementary Table S2). Based on the selection criteria of a minimum three or more independent identifications, we came to a list of 71 proteins. Of these, confirmed expression in human urine, based on the data from Human Protein Reference Database, had 29 proteins. As none of these proteins was expressed solely in the prostate and only the prostate-specific antigen (KLK3) was characterized with elevated expression in the prostate, compared to other tissues, further selection was performed based on the number of independent identifications by proteomics tissue studies, expression in normal and PCa tissues (HPA), molecular functions, biological processes (UniProtKB), and literature data mining (association with PCa/Ca). As a result, seven proteins were chosen for further evaluation in the urine of PCa patients and controls: alpha-2-glycoprotein 1, zinc binding (AZGP1), malate dehydrogenase 2 (MDH2), fatty acid binding protein 5, epidermal (FABP5), enolase 1 (ENO1), glutathione S transferase Pi (GSTP1), cytovillin/ezrin (EZR), and glutathione S-transferase Mu 2 (GSTM2).

GSTP1 and AZGP1 have been identified the most by proteomics tissue studies, with seven and five independent identifications with differential abundance in PCa, respectively. ENO1 has been identified by four studies, and the remaining EZR, FABP5, GSTM2, and MDH2 were identified by three studies. The observed regulation trend in PCa was increased abundance for ENO1, EZR, FABP5, and MDH2 and decreased abundance for GSTP1, GSTM2, and AZGP1. According to data from HPA, AZGP1 and MDH2 have shown high protein expression in both normal and tumor prostates, ENO1 and EZR have shown medium expression, GSTP1 had shown medium expression in normal prostate tissue, but medium to not detected in PCa, and FABP5 and GSTM2 were not detected in normal prostate tissue, while in PCa, they showed variable expression from medium to none. All seven proteins were implicated in prostate cancer development or progression, as well as in various other types of cancers by numerous proteomics, genomics, and functional studies (Supplementary Table S3). Three of these proteins, namely GSTP1, AZGP1, and GSTM2, are part of commercial tissue-based gene assays for PCa detection and stratification, such as ConfirmMDX and OncotypeDX.

### 3.2. Diagnostic Sensitivity and Specificity in PCa Detection

The preselected protein candidates were measured in urine samples from 85 patients of which the control group (BPH) and GS6 had 17 samples each, GS7 and GS9 had 18 samples each, and GS8 had 15 samples, respectively. The mean values (±SD) for serum PSA in the individual groups were as follows: 8.8 ± 4.8 ng/mL for the BPH, 9.7 ± 8.2 ng/mL for the GS6, 15.8 ± 13.6 ng/mL for the GS7, 28.9 ± 24.6 ng/mL for the GS8, and 80.1 ± 72.2 ng/mL for the GS9 group (Table 1). All patients were matched for age (group means 66.7–69.4 years).

**Figure 2:**
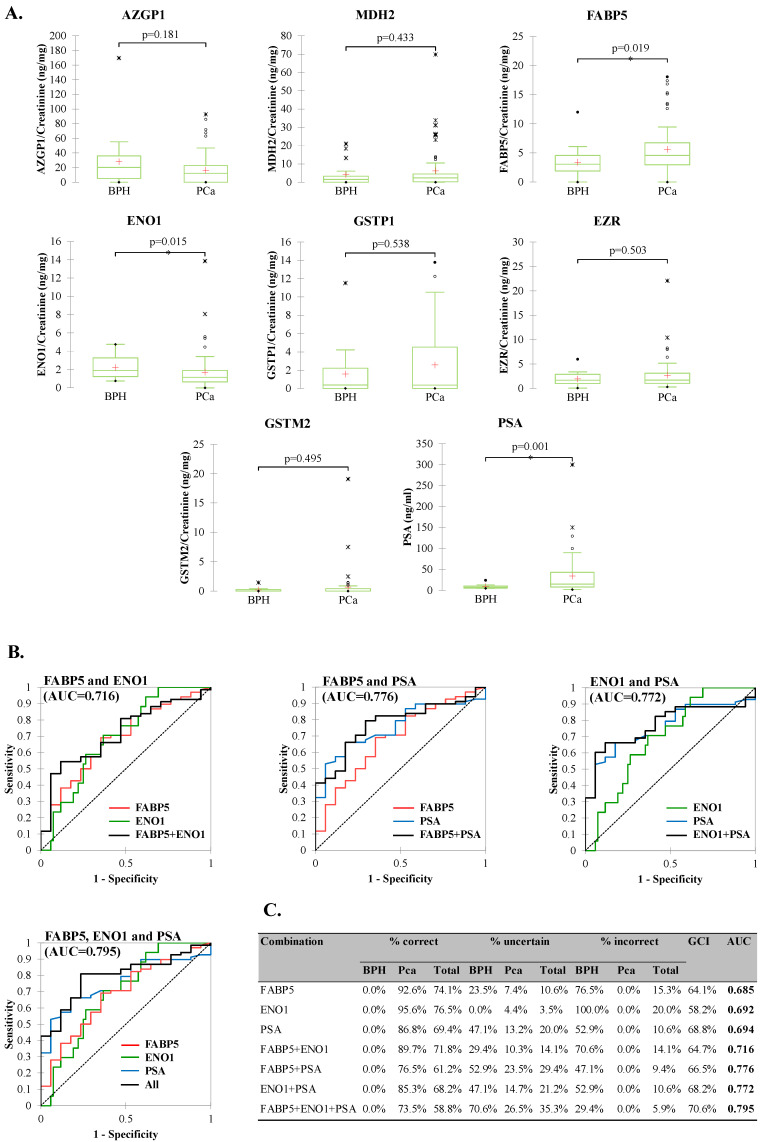
Diagnostic model development. (**A**) Levels of the investigated proteins and serum PSA in the BPH and PCa groups. In the box plot graphs, the median (−), 25th, and 75th percentiles, as well as the minimum/maximum (•), outliers (*), and mean (+), are presented. (**B**) Receiver operating characteristic (ROC) curves and (**C**) binary logistic regression predictive quality of combinations of FABP5, ENO1, and PSA for PCa detection.

To determine the diagnostic potential in detecting PCa, samples were grouped into two groups: BPH and PCa (GS6-9). As the normality testing of the obtained data showed that variables do not follow a normal distribution, statistically significant differences at the level of tested proteins between BPH and PCa were accessed by Mann–Whitney U-test. The results showed that a significant difference in protein levels between PCa and BPH showed FABP5 and ENO1, with FABP5 showing increased levels in PCa (*p* = 0.019) and ENO1 showing the opposite (*p* = 0.015). The levels of PSA also showed significant differences between the BPH and PCa groups (*p* = 0.001) (Figure 2A).

To determine the diagnostic potential of urinary FABP5 and ENO1, receiver operating characteristic (ROC) curves were used. The area under curve (AUC) results for FABP5 and ENO1 were 0.685 (95% CI: 0.544–0.825; *p* = 0.010) and 0.692 (95% CI: 0.568–0.816; *p* = 0.002), respectively. The optimal cutoffs for the proteins were: 3.25 ng FABP5/mg creatinine (64.7% specificity, 69.1% sensitivity) and 1.37 ng ENO1/mg creatinine (63.2% specificity, 70.6% sensitivity). The optimal cutoff of serum PSA was 10.6 ng/mL (82.4% specificity, 66.2% sensitivity), with an AUC of 0.694 (95% CI: 0.647–0.865; *p* < 0.0001). 

The binary logistic regression method applied to assess the diagnostic accuracy of different combinations of these proteins showed that the accuracy of the models increased with their combination, as well as with the combination of serum PSA (Figure 2B). FABP5 and ENO1 individually had the highest percentage of correctly classified samples (74.12% and 76.47%, respectively), compared with the rest of the combinations (Figure 2C).

However, as they also showed the highest percentage of incorrectly classified samples (15.29% and 20.00%, respectively), they had the medium goodness of classification index (GCI) of 64.12% and 58.24%, respectively. The PSA alone had a lower percentage of correctly classified samples (69.41%) than FABP5 or ENO1, which showed a higher GCI of 68.82%, due to the lower percentage of incorrectly classified samples. The combination of FABP5 and ENO1 lowered the percentage of correctly, as well as incorrectly, classified samples, increasing GCI to 64.71% and AUC to 0.716. The combination of all three proteins showed the highest specificity and sensitivity in discrimination between BPH and PCa, with AUC = 0.795 and GCI of 70.59%. Although this model showed the lowest percentage of correctly classified samples (58.82%) and the highest percentage of uncertainty, the percentage of incorrectly classified samples here was the lowest (5.88%), compared to the rest of the models.

The post-hoc power analyses for FABP5 and ENO1, based on the sample sizes of BPH (n = 17) and PCa (n = 68) groups, as well as the obtained variance within groups, gave the calculated powers of 0.869 and 0.360, respectively. The calculated power for FABP5 was above the generally accepted minimum power of 80%. For ENO1, the achieved power was low, mainly due to high variance in the PCa group. Based on this, the power of 0.8 was estimated to be obtained with a total sample size of 340.

### 3.3. Diagnostic Sensitivity and Specificity in PCa Progression

**Figure 3:**
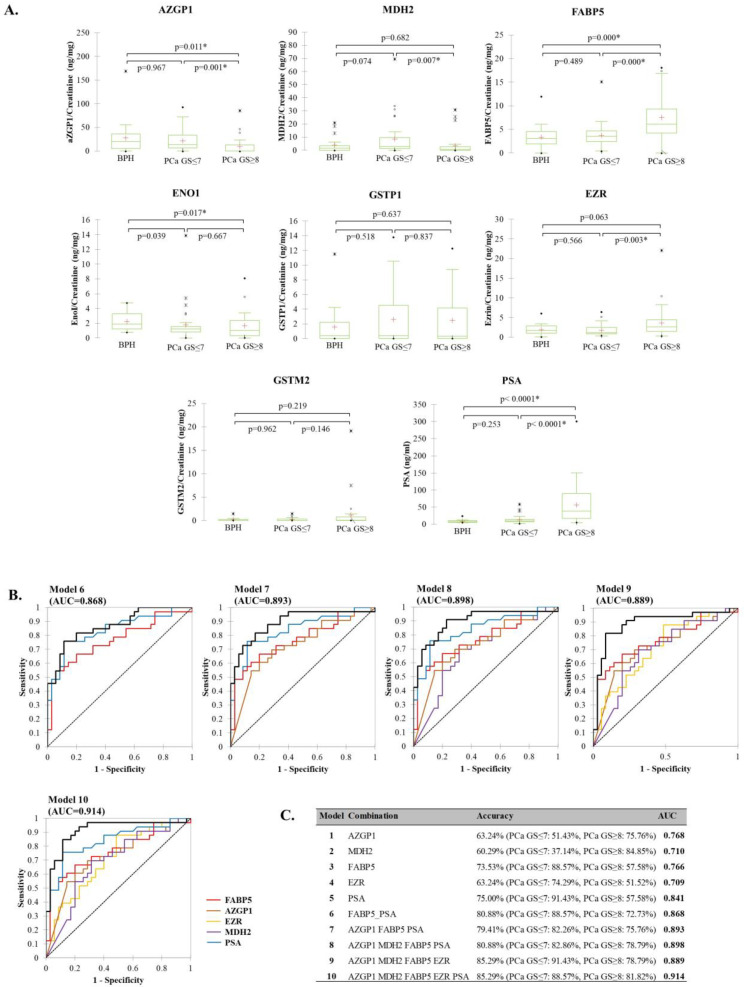
Diagnostic potential of the tested biomarkers for PCa progression: (**A**) Levels of the investigated proteins in low GS PCa (GS ≤ 7) and high GS PCa (GS ≥ 8) groups. In the box plot graphs, median (−), 25th, and 75th percentiles, as well as minimum/maximum (•), outliers (*), and mean (+), are presented; (**B**) Diagnostic accuracy in discrimination between low and high GS groups, depicted with ROC curves and (**C**) binary logistic regression outputs for the various combinations of AZGP1, MDH2, FABP5, EZR, and PSA.

To determine the diagnostic potential of the investigated urinary proteins in detecting advanced PCa, samples were grouped into three groups: BPH, low GS PCa (GS ≤ 7), and high GS PCa (GS ≥ 8). Differences at the level of tested proteins between tested groups were accessed by nonparametric Kruskal–Wallis, combined with Dunn’s test for multiple comparisons and Bonferroni correction of multiple testing. Statistically significant differences among groups showed AZGP1 (*p* = 0.003), MDH2 (*p* = 0.020), FABP5 (*p* < 0.0001), ENO1 (*p* = 0.047), EZR (*p* = 0.009), and PSA (*p* < 0.0001) (Figure 3A). From these, AZGP1 and ENO1 showed a steady decrease of levels from BPH to low GS PCa, with the lowest in high GS PCa patients, while FABP5 and PSA showed the opposite. 

Statistically significant differences between low and high GS groups showed AZGP1 (*p* = 0.001), MDH2 (*p* = 0.007), FABP5 (*p* < 0.0001), EZR (*p* = 0.003), and PSA (*p* < 0.0001). To determine the diagnostic potential of these proteins, ROC curves analysis was used. The highest individual accuracy was observed for FABP5 and AZGP1, with AUC = 0.766 (95% CI: 0.651–0.882; *p* = < 0.0001) and 0.768 (95% CI: 0.606–0.846; *p* < 0.001), respectively. The optimal cutoffs for the proteins were: 4.94 ng FABP5/mg creatinine (80.0% specificity, 66.7% sensitivity) and 11.86 ng AZGP1/mg creatinine (71.4% specificity, 69.7% sensitivity). MDH2 and EZR showed lower AUC of 0.710 (95% CI: 0.564–0.817; *p* = 0.003) and 0.709 (95% CI: 0.586–0.832; *p* = 0.001), respectively, with optimal cutoffs of 2.41 ng MDH2/mg creatinine (68.6% specificity, 69.7% sensitivity) and 1.21 ng EZR/mg creatinine (51.4% specificity, 87.9% sensitivity). The optimal cutoff of serum PSA was 17.0 ng/mL (88.6% specificity, 75.8% sensitivity), with an AUC of 0.841 (95% CI: 0.743–0.936; *p* < 0.0001). 

Diagnostic accuracy in discrimination between low and high GS patients of these individual proteins and their combinations was determined by logistic regression analysis (Figure 3B,C). Among individual proteins, the highest accuracy was observed for FABP5 and PSA, respectively. The diagnostic model based on FABP5 correctly classified 73.53% of total cases (PCa GS ≤ 7: 88.57%, PCa GS ≥ 8: 57.58%)). The model based on PSA alone correctly classified 75.00% of total cases, due to the higher percentage of correct classification of the PCa GS ≤ 7 group (PCa GS ≤ 7: 91.43%, PCa GS ≥ 8: 57.58%)). The combination of all proteins showed increased accuracy (AUC = 0.889), compared to PSA alone (AUC = 0.841), with 85.29% of total correct classification and 91.43% and 78.79% correct classification of PCa GS ≤ 7 and PCa GS ≥ 8 groups, respectively. The model with the highest accuracy (AUC = 0.914) combined all proteins and PSA. The model correctly classified 85.29% of total samples with 88.57% of the PCa GS ≤ 7 group and 81.82% of the PCa GS ≥ 8 group.

The post-hoc power analyses calculated the power for AZGP1, MDH2, FABP5, and EZR to be 0.9302, 0.7340, 0.9996, and 0.9141, respectively. Overall, for AZGP1, FABP5, and EZR the sample size used in this study was sufficient to obtain high power over 90%. In the case of MDH2, the calculated power is slightly below the generally accepted minimum power of 80%, due to higher variance in both groups. 

### 3.4. Correlation between Tested Biomarkers and Clinical Parameters

**Figure 4:**
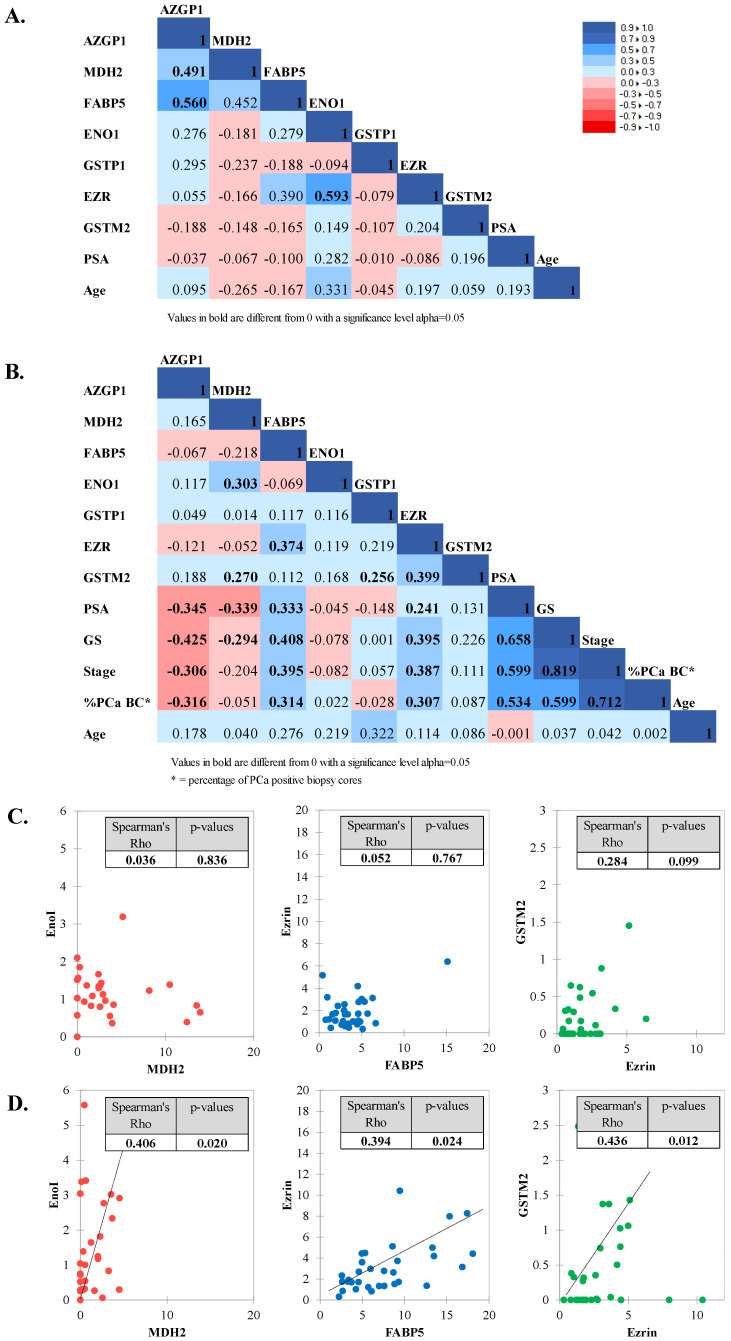
Spearman correlation between tested biomarkers and clinical parameters in (**A**) BPH cohort; (**B**) PCa cohort. The correlation pattern between ENO1, MDH2, FABP5, EZR, and GSTM2 in low GS PCa cohort (**C**) and high GS PCa cohort (**D**).

Correlation analysis using Spearman correlation was performed to investigate whether the urine levels of the biomarkers were clinically independent prognostic factors for PCa. In the control BPH cohort, AZGP1 showed significant low positive correlation with MDH2 (r = 0.491; *p* = 0.048) and moderate positive correlation with FABP5 (r = 0.560; *p* = 0.021) (Figure 4A). A moderate positive correlation was also observed between ENO1 and EZR (r = 0.593; *p* = 0.014).

In the PCa cohort, there were several significant low positive correlations between proteins: ENO1 with MDH2 (r = 0.303; *p* = 0.012); FABP5 with EZR (r = 0.374; *p* = 0.002) and GSTM2 with EZR (r = 0.399; *p* = 0.001) (Figure 4B). After analyzing these correlations in low (Figure 4C) and high GS PCa (Figure 4D) sub-cohorts, it was obvious that the observed correlations were significant for high GS PCa only. 

None of the proteins in the BPH cohorts correlated with age and serum PSA (Figure 4A). In the PCa cohort also, none of the proteins showed a significant correlation with age (Figure 4B). On the other hand, several proteins in the PCa group showed a significant low positive or negative correlation with serum PSA and clinical parameters. AZGP1 showed significant low negative correlation with PSA (r = −0.345; *p* = 0.004), GS (r = −0.425; *p* < 0.001), stage (r = −0.306; *p* = 0.011), and percentage of positive biopsy cores (r = −0.316; *p* = 0.009). MDH2 showed also significant low negative correlation with PSA (r = −0.339; *p* = 0.005) and GS (r = −0.294; *p* < 0.015). On the other hand, FABP5 and EZR showed a significant low positive correlation with PSA (r = 0.333; *p* = 0.006 and r = 0.241; *p* = 0.048, respectively), GS (r = 0.408; *p* < 0.001 and r = 0.395; *p* = 0.001, respectively), stage (r = 0.395; *p* = 0.001 and r = 0.387; *p* = 0.001, respectively), and percentage of positive biopsy cores (r = 0.314; *p* = 0.009 and r = 0.307; *p* = 0.011, respectively). Serum PSA was moderately positively correlated with GS (r = 0.658; *p* < 0.0001), stage (r = 0.599; *p* < 0.0001), and percentage of positive biopsy cores (r = 0.534; *p* < 0.0001) in the PCa cohort. In addition, GS showed a high positive correlation with the stage (r = 0.819; *p* < 0.0001) and a moderate positive correlation with the percentage of positive biopsy cores (r = 0.599; *p* < 0.0001). The stage was also highly positively correlated with the percentage of positive biopsy cores (r = 0.712; *p* < 0.0001).

## 4. Discussion

Although a lot of effort has been put, so far, into designing new molecular tools that will improve the clinical management of PCa, the currently available tests are still far from providing clear, precise diagnosis and risk stratification. Therefore, the identification of new molecular marker(s) that can be used as a non-invasive tool in the precise diagnosis and stratification of PCa patients remains a pertinent clinical need.

In an effort to support the implementation of new non-invasive biomarkers into practice, we have set up this study to test several potential biomarkers identified by comparative proteomics studies of prostate tissue. In the search for biomarker candidates, we have focused solely on prostate tissue-based studies for two main reasons: (1) prostate tissue is the direct site of molecular alterations implicated in cancer onset and progression and, therefore, the best source of potential PCa biomarkers for disease detection, stratification, and therapy; (2) the analysis of tissue material (as a complex mixture of prostate cells, immune and inflammatory cells, blood vessel cells, and fibroblasts) allows for the detection of the tumor proteome and/or in vivo secretome alterations created by host-tumor cell interaction that may be crucial factors for tumors to undergo progression or regression [42].

The selected tissue biomarkers in this study have well-known associations with PCa and some have well-recognized roles in cancer, in general. Alpha-enolase (ENO1) is one of the three enolase isoenzymes found in mammals, which functions as a glycolytic enzyme and as a structural lens protein (tau-crystallin) in the monomeric form. ENO1 overexpression and post-translational modifications were observed to have diagnostic and prognostic value in many cancer types [43]. ENO1 plays several roles in regard to cancer growth modulation, such as catalyzing glycolysis, maintaining mitochondrial membrane stability, regulating signaling pathways, and reorganizing the cytoskeleton, as well as binding plasminogen when expressed on the surface, a process exploited by cancer cells to promote metastasis, migration, and invasion [44]. ENO1 has been proposed as a PCa biomarker by numerous proteomics (discussed below in detail), genomics, and functional studies, as well as a biomarker for glioma, neurocytoma, cholangiocarcinoma, lung, kidney, nasopharyngeal cancer, etc. (detailed description is given in Supplementary Table S3).

Ezrin (EZR) is involved in cell network regulation by linking the actin cytoskeleton to the cell membranes and by controlling signal transduction by interaction with adhesion molecules and various growth factor receptors [45]. In cancer cells, the relative membrane localization of EZR is increased, which facilitates the process of cancer progression and invasion [46]. As such, it has been implicated as a biomarker in various human cancers, such as prostate, lung, breast, ovarian, cervical, and gastric cancer (Supplementary Table S3). 

Zinc-alpha-2-glycoprotein (AZGP1) is a secretory protein, with a strong affinity for zinc and fatty-acid binding, that exerts a role of a lipid-mobilizing adipokine involved in fat loss by lipid degradation in adipocytes [47]. AZGP1 has been demonstrated to inhibit cancer cell proliferation and invasion by inhibiting TGF-β-mediated epithelial–mesenchymal transition, based on which was recognized as a tumor suppressor [48]. AZGP1 is one of the most identified candidate biomarkers for PCa by numerous studies, as well as for bladder, colorectal, endometrial, pancreatic, head and neck cancer, and many others (Supplementary Table S3). 

In the initial selection of the biomarkers, we have included two members from the glutathione S-transferases family: glutathione S-transferase Pi 1 (GSTP1) and glutathione S-transferase Mu 2 (GSTM2). Glutathione S-transferases are a family of proteins involved in phase II of the detoxification process by catalyzing the conjugation of many hydrophobic and electrophilic endogenous and exogenous substances with reduced glutathione. GSTP1 is a recognized tumor suppressor that interacts and modulates several pathways involving cell growth, differentiation, and cell death, and as such, its association with cancer has been vast, featuring a plethora of studies linking its polymorphisms and hypermethylation with cancer [49]. The epigenetic regulation of GSTM2 has been associated with several cancers, among which is PCa, although there are far less studies compared to GSTP1, and far more variable findings in the context of proteomics PCa studies (Supplementary Table S3).

Fatty acid binding protein 5 (FABP5) belongs to a class of intracellular lipid-binding proteins that help in the transportation of lipids through the cellular compartments. Although ubiquitously expressed, its dysregulation is frequently observed in PCa. One of the possible mechanisms that explain this is its role in the transport of retinoic acid to its alternate intracellular receptor, PPAR-β/δ, which facilitates the transcription of genes associated with cell survival and proliferation [50]. In addition to PCa, FABP5 has been suggested as a biomarker for bladder and oral cancer, while other members of the family have been found frequently up-regulated in various cancers (Supplementary Table S3).

Malate dehydrogenase 2 (MDH2) is a mitochondrial enzyme involved in the Krebs cycle. MDH2 has been implicated as a PCa biomarker by several proteomics, genomics, and functional studies, although its role in cancer, and specifically in PCa, has not been explained so far.

Protein measurements in urine showed that only ENO1 and FABP5, out of all seven biomarkers, showed statistically significant differences between the BPH and PCa groups. The significant up-regulation of FABP5 in urine was equivalent to the observed up-regulation in tissue samples of PCa, compared to BPH, in several proteomics studies [22,30,51,52]. On the other hand, ENO1 showed down-regulation in the urine of PCa patients. This is opposite to the observed trend in tissue studies [29,37], but well in concordance with the observed down-regulation in the urine of PCa patients, by several comparative proteomics studies [53,54,55]. The observed accuracies of the diagnostic models based on individual proteins were similar to the accuracy of serum PSA. However, it should be stressed that the reported diagnostic accuracy of PSA was under the optimal cutoff of 10.6 ng/mL, which was way higher than the cutoff used in diagnostic purposes. In our cohort, 4 ng/mL serum PSA provided 0.0% specificity and 94.1% sensitivity. Models based on the combination of FABP5 and ENO1, as well as their combinations with PSA provided increased accuracy over the models based on individual proteins only. The highest accuracy in PCa detection provided the combined model of FABP5, ENO1, and PSA with AUC = 0.795. Further investigation of the correlation between the urine concentration of FABP5 and ENO1 and serum concentration of PSA among patients with PCa or BPH revealed no significant correlation. This suggests that FABP5 and ENO1 are independent PCa biomarkers, and these findings are well-deserved to be explored further in larger cohorts of patients.

Next, the evaluation of the diagnostic potential of the investigated urinary proteins in detecting advanced PCa revealed that statistically significant differences between low and high GS groups showed FABP5, EZR, AZGP1, and MDH2. 

FABP5 showed no significant difference between BPH and low GS group, but highly significant difference between low GS and high GS groups. These observations were supported by a previous study where FABP5 could differentiate between lymph node metastatic and localized PCa [34], as well as a study where it was detected as up-regulated in extracellular vesicles extracted from urine of patients with high Gleason score PCa [56]. Similarly, EZR also showed the same trend as FABP5. These observations were, in particularly, well-aligned with previous studies, where, in addition to detecting up-regulated EZR in PCa tissue and urine, compared to BPH [23,57], there was plenty of evidence for significantly overexpressed EZR in high-grade prostatic intraepithelial neoplasia, compared to PCa in a less aggressive stage [34,58,59]. AZGP1, on the other hand, showed significant down-regulation in the urine of high GS, compared to low GS. This was well in concordance with its proposed role of tumor suppressor, backed up primarily by gene expression studies where low expression of AZGP1 is associated with a higher risk of aggressive time-dependent outcomes in PCa [60,61,62,63]. Proteomics studies analyzing tissue have observed the same down-regulation on the protein level in PCa, compared to BPH [22,23], as well as in high vs low aggressive disease [20,32,64]. However, there are also proteomics studies that have observed the opposite trend of AZGP1 in tissue [39], seminal plasma [65], serum [66], and urine of PCa patients, showing predictive power as a solo and as a panel biomarker, together with PSA [67]. In regard to MDH2, we have observed an increase, although not significantly in low GS, compared to BPH, and a significant decrease in high GS, compared to low GS. This observation was in line with previous proteomics studies of PCa, where MDH2 was found to be up-regulated in PCa tissue [26,29,37], as well as significantly down-regulated in aggressive, compared to low-risk PCa [25,68].

Surprisingly, GSTP1 and GSTM2 did not show significant changes with PCa progression in our setting. All of the proteomics studies so far that have detected GSTP1 [23,26,28,29,30,69] and GSTM2 [22,28] as significantly dysregulated in PCa were analyzing tissue and all have come to a uniform conclusion, i.e., that these two Glutathione S-transferases were down-regulated in PCa tissue. In addition, many studies show, on the transcript level, that epigenetic silencing of GSTP1 and GSTM2 is a molecular marker for PCa, both for the diagnosis and progression of the disease (Supplementary Table S3). Nevertheless, more studies similar to ours are needed to establish a relevant conclusion, in regard to glutathione S-transferases protein levels in the urine of PCa patients.

The combination of FABP5, EZR, AZGP1, and MDH2 showed the highest diagnostic accuracy and correctly classified 85.29% of PCa samples into low and high GS groups. This was far more than the individual accuracy of the proteins or accuracy based on PSA. FABP5 and AZGP1 contributed the most in the prognostic biomarker panel, with FABP5 alone showing the highest percentage of 88.57% of correctly classified samples from the low GS group and AZGP1 showing the highest percentage of 75.76% of correctly classified samples from high GS group. Furthermore, the test based on these four proteins could discriminate non-aggressive from aggressive PCa, independent of PSA. The addition of PSA to the panel did not change the accuracy, in terms of correctly classified PCa samples. The significant correlation of these four proteins with PCa was also evident from the correlation analysis with the clinical data. AZGP1, FABP5, and EZR showed significant a correlation with GS, stage, and percentage of positive biopsy cores. MDH2 also showed a significant correlation with GS, but failed to do so with the remaining clinical features. We speculate that this could be due to the relatively small sample cohort, but this remains to be proven in the future. Moreover, each of these proteins represent an independent PCa biomarker, as they do not significantly correlate with each other, with the exception of FABP5, which showed a significant low positive correlation with EZR (r = 0.394; *p* = 0.024), but only in the high GS group. 

The strength of this study is in its concept of high-quality data mining of non-hypothesis driven proteomics comparative PCa studies. Independent identification of the same candidate biomarkers in independent cohorts and by independent research groups and techniques holds great promise, in regard to the credibility of the findings. In addition, the large-scale disease spectrum (BPH and the low and high-risk localized, as well as progressed stages of the disease) and very well clinically and histopathologically defined cohort represents another strength of the study. The limitations are the relatively small sample cohort, potential variations in the urine collection and storage prior to the delivery to our lab, and inherited variability of the chosen technique for measurement of the proteins in urine. However, it is worth stressing that the post-hoc power analyses confirmed that, for all proposed biomarkers, with exception of ENO1, we can confidently conclude that this study was sufficiently powered, in terms of the size of the investigated cohort, to answer the main aims/questions put forth at the beginning of the study design.

We believe that the developed biomarker panels for PCa detection and prognosis in this study hold great promise as a new tool in PCa diagnostic management. However, precise quantification, as well as the assessment of the protein intra- and inter-variability in urine, is a critical step toward clinical application. Validation using larger patient cohorts with PCa, as well as other tumor types, will be necessary to more precisely establish the value of the proposed biomarker panels for non-invasive PCa detection and prognosis.

## 5. Conclusions

In this study, we presented, for the first time, a systematic analysis of the diagnostics and prognostic PCa biomarkers identified by comparative proteomics studies on human prostate tissues. The validation of the seven preselected candidate biomarkers revealed a clinically relevant correlation between the urine concentrations of five of those proteins and PCa. The observed relationship proposes two new biomarker panels for non-invasive diagnosis and prognosis of PCa. In addition, this study opens a way to further test and validate more high-quality proteomics biomarkers that could ultimately add value to the clinical management of PCa.

## Figures and Tables

**Table 1:** Clinical and histopathological data of patients included in the study.

Group	Diagnosis	Patients per Group	Age (Mean ± SD)	Age (Median)	PSA (Mean ± SD)	Serum PSA (Median)	Gleason Score	Histopathological Stage			Stage
								T	N	M	
Control	Benign prostate hyperplasia	17	68.9 ± 6.5	68.0	8.8 ± 4.8	7.6	/	/	/	/	/
GS6	Prostate cancer (Gleason score = 6)	17	66.7 ± 5.8	68.0	9.7 ± 8.2	7.6	(3 + 3)	T2-T3a	Nx-N0	Mx	I–IIIB
GS7	Prostate cancer (Gleason score = 7)	18	67.7 ± 4.6	68.5	15.8 ± 13.6	11.7	(3 + 4) (4 + 3)	T2c-T4b	Nx-N0	Mx	II–IIIB
GS8	Prostate cancer (Gleason score = 8)	15	69.4 ± 7.1	70.0	28.9 ± 24.6	19.3	(3 + 5) (4 + 4)	T3b-T4	N0-N1	Mx	III–IV
GS9	Prostate cancer (Gleason score = 9)	18	67.7 ± 6.0	67.0	80.1 ± 72.2	66.5	(4 + 5) (5 + 4)	T2b-T4	N0-N1	Mx	III–IV

## Data Availability

The data presented in this study are available on request from the corresponding author.

## Supplementary Materials

The following supporting information can be downloaded at: https://www.mdpi.com/article/10.3390/diagnostics12123184/s1, Supplementary Table S1: Age, clinical, and histopathology information of the patients included in the study (XLSX). Supplementary Table S2: Study design with criteria for the selection of PCa biomarker candidates for independent validation with ELISA in the urine of patients and controls (XLSX). Supplementary Table S3: Functional characterization and association with prostate and other types of malignancy of the selected biomarkers (DOCX).
