# Supplementary material for: Potential Role of Seven Proteomics Tissue Biomarkers for Diagnosis and Prognosis of Prostate Cancer in Urine
Source: Diagnostics (Basel). 2022 Dec 16;12(12):3184. doi: 10.3390/diagnostics12123184 (PMC9777474; doi:10.3390/diagnostics12123184)
Supplement: Supplementary file 1 [file diagnostics-12-03184-s001.zip › Supplementary Table S3_final.pdf]

Supplementary Table S3: Functional characterization and association with prostate and other types of malignancy of the selected biomarkers.

Protein name	Gene Name	Molecular/biological function(s)	Classification and involvement in disease	Association with PCa (proteomics studies)	Associations with PCa (genomics and functional studies)	Association with cancer
Alpha-enolase	ENO1	Alpha-enolase is one of three enolase isoenzymes found in mammals. It functions as a glycolytic enzyme and as a structural lens protein (tau-crystallin) in the monomeric form. Alternative splicing of this gene results in a shorter isoform that has been shown to bind to the c-myc promoter and function as a tumor suppressor.	ENO1 overexpression and post-translational modifications could be of diagnostic and prognostic value in many cancer types [1].	ENO1 showed down-regulation in exosomes derived from PCa cells compared to normal cells [2];<br>Stromal ENO1 levels are increased in PCa compared with those in normal tissue [3];<br>Down-regulated in urine from PCa patients [4];<br>Down-regulated in urine from PCa patients compared to BPH [5];<br>Decreased methylation and increased expression of ENO1 have been detected in prostate cancer tissues [6];<br>ENO1 is up-regulated in prostate cancer tissue [7];<br>Anti-ENO1 antibodies are increased in serum from prostate cancer patients [8];<br>ENO1 is up-regulated in extracellular vesicles (serum) from PCa patients after acquiring cabazitaxel resistance [9];	Over-expression of ENO1 modulates the MAPK pathway by inhibiting MEK5 and inhibiting prostate cancer growth [10];<br>ENO1 knock-down results with reduced cell cycle progression in prostate cancer cells [11];	ENO1 mRNA and protein levels were upregulated in glioma tissues [12];<br>Upregulated in lung carcinoma [13-15];<br>Slightly elevated in serum and urine of renal cell carcinoma patients [16];<br>Down-regulation of ENO1 in nasopharyngeal carcinoma [17];<br>Upregulated in serum in cholangiocarcinoma [18];<br>ENO1 possesses tumor-suppressing effects in neurocytoma [19];
Fatty acid binding protein 5	FABP5	The fatty-acid-binding proteins (FABPs) are a family of carrier proteins for fatty acids and other lipophilic substances such as eicosanoids and retinoids. These proteins are thought to facilitate the transfer of fatty acids between extra- and intracellular membranes. FABPs roles include fatty acid uptake, transport and metabolism.	Involved in prostate cancer cell growth [20];<br>Novel molecular target in prostate cancer [21];	Up-regulated in tissue from prostate cancer patients [22];<br>FABP5 has been found to be up-regulated in tissue from prostate cancer patients with different racial disparities [23];<br>FABP5 is upregulated in prostate cancer tissue compared to BPH [24];<br>FABP5 is upregulated in PCa tissues compared to BPH and normal tissues [25];<br>FABP5 is up-regulated in urine extracellular vesicles from patients with high Gleason score PCa [26];<br>FABP5 has been found to be up-regulated in prostate cancer tissue, and could differentiate between lymph node metastatic PCa and localized from [27];	Up-regulated gene expression in prostate cancer tissue [28];<br>FABP5 gene expression was not linked to prostate cancer's clinical outcome but is associated with PPAR signaling, suggesting a drugable nature [29];<br>Co-expression of FABP5 with FASN and MAGL is associated with lipid-mediated metastasis [30];<br>CNFI26 is a potent FABP5 inhibitor [31];<br>Inactivated FABP5 suppresses malignant progression of prostate cancer cells [32];	Up-regulated in bladder squamous cell carcinomas tissue [33];<br>Up-regulated in bladder cancer urine [34];<br>Down-regulated FABP5 is associated with metastasis of squamous cell carcinoma of the oral tongue [35];<br>**Family members of FABP5**:<br>Up-regulated in endometrial carcinoma [36];<br>Ubiquitous overexpression in endometrial cancers [37];<br>Up-regulated in breast cancer [38];<br>Up-regulated in PCa [25];<br>FABP family involved in cancer [39];

Alpha-2-glycoprotein 1, zinc-binding	AZGP1	It is a secretory protein, with a structure similar to the MHC I molecules with strong affinity for zinc and fatty-acid binding. It exerts a role of lipid-mobilizing adipokine involved in fat loss by lipid degradation in adipocytes [40].	Heavily involved in cachexia, a wasting syndrome associated with cancer [41];<br>AZGP1 has been found to inhibit cancer invasion by inhibiting TGF-β mediated epithelial-mesenchymal transition, facilitating its role as a tumor suppressor [42];	AZGP1 is down-regulated in PCa tissue compared to BPH [43];<br>AZGP1 has been found to be up-regulated in tissue from PCa patients [44];<br>AZGP1 is up-regulated in seminal plasma of PCa patients [45];<br>AZGP1 is up-regulated in serum but did not show a similar pattern in tissues of PCa patients [46];<br>AZGP1 is up-regulated in urine of PCa patients, showing predictive power as a solo and as a panel biomarker together with PSA[47];<br>AZGP1 shows a down-regulated trend in epithelial prostate cancer cells, and negative correlation with Gleason grading [48];<br>AZGP1 is down-regulated in tissue from PCa patients [24];<br>AZGP1 is down-regulated in aggressive compared to non-aggressive PCa tissues [49, 50];	Low AZGP1 expression was associated with metastasis or death from prostate cancer [51];<br>Up-regulated AZGP1 gene expression is associated with decreased risk of PCa recurrence [52];<br>Low expression of AZGP1 is an independent predictor of recurrence in margin-positive localized PCa [53];<br>Through meta-analysis, low expression of AZGP1 is associated with a higher risk of aggressive time-dependent outcomes in PCa patients undergoing radical prostatectomy [54];	Up-regulated in urine of bladder cancer patients [55];<br>Up-regulated in serum of colorectal cancer patients [56];<br>Up-regulated in urine of endometrial cancer patients [57];<br>Up-regulated in multiple myeloma [58];<br>Up-regulated in cancer cachexia in human urine [59];<br>Up-regulated in saliva in head and neck squamous cell carcinoma patients [60];<br>Tumor suppressor in pancreatic cancer [42];
Glutathione S-transferase mu 2 (muscle)	GSTM2	Glutathione S-transferases comprise a family of enzymes that are critical for the inactivation of toxins and carcinogens. Glutathione S-transferase mu2 is a phase II detoxification enzyme. Proposed role in cancer as tumor suppressor.	GSTM2 is associated with metabolism of xenobiotics, such as carcinogens, catalyzing the conjugation of these compounds with glutathione [61];<br>Glutathione S-transferases have been associated with resistance to anticancer drugs [62] and have been shown to have a regulatory role in the proliferation and differentiation of tumor cells [63];	GSTM2 has been shown to be down-regulated in tissue from prostate cancer patients [24];<br>GSTM2 has been shown to be down-regulated in tissue from prostate cancer patients [64];	Loss of expression of all GST subclasses results in the progression of prostatic neoplasia to carcinoma [65];<br>Epigenetic silencing of GSTM2 is a molecular marker for PCa [66];<br>Simultaneous genetic silencing of GSTM2 and MYCL2 is proposed as a molecular marker panel to predict biochemical recurrence of PCa after radical prostatectomy of high-risk localized PCa [67];<br>Multigene panel, consisting of GAS6, GSTP1 and HAPLN3, has been created and validated for the classification of benign and malignant PCa [68];	GSTM2 differential expression in breast cancer cells [69];<br>Low expression of GSTM2 in lung cancers is due to hypermethylation of its promoter [70];<br>Association with kidney and bladder tumors [71];

Glutathione S-transferase Pi 1	GSTP1	GSTP1 is thought to function in xenobiotic metabolism and play a role in susceptibility to cancer and other diseases.	Glutathione S-transferases play tumor suppressive, cyto-protective roles and interact and modulate several pathways involving cell growth, differentiation and cell death and as such are interconnected in the process of tumorigenesis [72];	GSTP1 is down-regulated in PCa tissue compared to BPH (Garbis, 2008)<br>GSTP1 has been found as down-regulated in tissue biopsy from PCa patients [22];<br>GSTP1 is down-regulated in PCa tissue compared to benign prostate hyperplasia [73];<br>GSTP1 has been shown to have a down-regulated expression in prostate cancer tissue [74];<br>GSTP1 is down-regulated in prostate cancer tissue [64];<br>GSTP1 is down-regulated in prostate cancer tissue [6];	The promotor methylation of GSTP1 have high implications in early PCa initiation and development [75];<br>Hyper methylation of the GSTP1 promotor leads to silencing in PCa [76];<br>GSTP1 is part of a gene panel used in urine for non-invasive detection and risk stratification for PCa [77];<br>GSTP1 hyper methylation can be used for selecting PCa patients for active surveillance [78];<br>ProCUrE, a non-invasive urinary methylation assay, consisting of LASSO, HOXD3 and GSTP1 is can assist in the early detection and prognosis of PCa [79];<br>GSTP1 methylation in combination with 4 other genes can help with risk assessment of PCa [80];<br>GSTP1 methylation can predict missed PCa [81];<br>GSTP1 has been shown to be hyper methylated selectively in PCa tissue [82];	Hyper methylation of GSTP1 has been shown in:<br>lung cancer [83];<br>breast cancer [84];<br>hepatocellular carcinoma [85];<br>bladder cancer [86];
Malate dehydrogenase 2	MDH2	The protein encoded by this gene is localized to the mitochondria and may play pivotal roles in the malate-aspartate shuttle that operates in the metabolic coordination between cytosol and mitochondria.	As a mitochondrial enzyme, MDH2 has an association with mitochondrial diseases such as early-onset severe encephalopathy [87];	MDH2 is up-regulated in PCa tissue [7];<br>MDH2 is down-regulated in highly metastatic variant LNCaP-LN3 cell line [88];<br>MDH2 is up-regulated in PCa tissue, that continues to increase with the cancer progression [6];<br>MDH2 has been detected as up-regulated in prostate cancer tissue both with a proteomic approach and a gene expression microarray [74]; MDH2 is down-regulated in PCa patients which experience biochemical relapse compared to patients who do not [89];	MDH2 overexpression decreases the relapse-free period of prostate cancer patients. MDH2 knockdown in prostate cancer cell lines increases sensitivity to docetaxel [90];<br>Treatment with Alternol results in reduced cell respiration and ATP production in PCa cells [91];	MDH2 up-regulation is associated with poor responses to radiation treatment in colorectal cancer [92];
Ezrin	EZR	As a member of the Ezrin/Radixin/Moesin protein family, this protein serves as an intermediate between the plasma membrane and the actin cytoskeleton.	Ezrin (EZR) is involved in cell network regulation by linking the actin cytoskeleton to the cell membranes and also control signaling transduction by interacting with adhesion molecules and various growth factor receptors [93];	EZR is up-regulated in PCa tissue compared to BPH [43];<br>EZR overexpression has been correlated with PCa and adverse prognosis [95];<br>EZR has been found as up-regulated in tissues from lymph node metastatic PCa compared to localized PCa [27];	EZR is involved in androgen-induced cell invasion [98];<br>EZR in MCF7 and PC3 were shown to form a complex with podocalyxin, which implicates its role in aggressive forms of prostate and breast cancer [99];<br>c-Myc regulates EZR expression in the presence of androgens, inducing cell invasion [100];	EZR has been associated with several types of cancer such as: lung cancer [103], breast cancer [104], ovarian cancer [105], cervical cancer [106], gastric cancer [107].

			In cancer cells, the relative membrane localization of EZR is increased which facilitates the process of cancer progression and invasion [94];	EZR is up-regulated in urine-derived extracellular vesicles from PCa patients [96];<br>EZR has been detected as overexpressed in high-grade prostatic intraepithelial neoplasia compared to PCa in a less aggressive stage [97];	EZR is linked with modification of cellular adhesion and cellular motility [101];<br>EZR hadn't shown differential gene expression in prostate cancer compared to benign tissue [102];	

REFERENCES

1. P. Cappello, M. Principe, S. Bulfamante,F. Novelli, Alpha-Enolase (ENO1), a potential target in novel immunotherapies, Front Biosci (Landmark Ed) 22 (2017) 944-959.
2. D. Duijvesz, K.E. Burnum-Johnson, M.A. Gritsenko, A.M. Hoogland, M.S. Vredenbregt-van den Berg, R. Willemsen, T. Luider, L. Pasa-Tolic,G. Jenster, Proteomic profiling of exosomes leads to the identification of novel biomarkers for prostate cancer, PloS one 8 (2013) e82589.
3. L. Yu, J. Shi, S. Cheng, Y. Zhu, X. Zhao, K. Yang, X. Du, H. Klocker, X. Yang,J. Zhang, Estrogen promotes prostate cancer cell migration via paracrine release of ENO1 from stromal cells, Mol Endocrinol 26 (2012) 1521-30.
4. I. Rehman, A.R. Azzouzi, J.W. Catto, S. Allen, S.S. Cross, K. Feeley, M. Meuth,F.C. Hamdy, Proteomic analysis of voided urine after prostatic massage from patients with prostate cancer: a pilot study, Urology 64 (2004) 1238-43.
5. K. Davalieva, S. Kiprijanovska, I. Maleva Kostovska, S. Stavridis, O. Stankov, S. Komina, G. Petrusevska,M. Polenakovic, Comparative Proteomics Analysis of Urine Reveals Down-Regulation of Acute Phase Response Signaling and LXR/RXR Activation Pathways in Prostate Cancer, Proteomes 6 (2017)
6. L. Latonen, E. Afyounian, A. Jylha, J. Nattinen, U. Aapola, M. Annala, K.K. Kivinummi, T.T.L. Tammela, R.W. Beuerman, H. Uusitalo, M. Nykter,T. Visakorpi, Integrative proteomics in prostate cancer uncovers robustness against genomic and transcriptomic aberrations during disease progression, Nat Commun 9 (2018) 1176.
7. S. Skvortsov, G. Schafer, T. Stasyk, C. Fuchsberger, G.K. Bonn, G. Bartsch, H. Klocker,L.A. Huber, Proteomics profiling of microdissected low- and high-grade prostate tumors identifies Lamin A as a discriminatory biomarker, J Proteome Res 10 (2011) 259-68.
8. T.W. Sanchez, G. Zhang, J. Li, L. Dai, S. Mirshahidi, N.R. Wall, C. Yates, C. Wilson, S. Montgomery, J.Y. Zhang,C.A. Casiano, Immunoseroproteomic Profiling in African American Men with Prostate Cancer: Evidence for an Autoantibody Response to Glycolysis and Plasminogen-Associated Proteins, Mol Cell Proteomics 15 (2016) 3564-3580.
9. S. Hishida, K. Kawakami, Y. Fujita, T. Kato, M. Takai, K. Iinuma, K. Nakane, T. Tsuchiya, T. Koie, Y. Miura, M. Ito,K. Mizutani, Proteomic analysis of extracellular vesicles identified PI3K pathway as a potential therapeutic target for cabazitaxel-resistant prostate cancer, Prostate 81 (2021) 592-602.
10. A.K. Ghosh, R. Steele,R.B. Ray, c-myc Promoter-binding protein 1 (MBP-1) regulates prostate cancer cell growth by inhibiting MAPK pathway, J Biol Chem 280 (2005) 14325-30.
11. A.K. Ghosh, R. Steele,R.B. Ray, Knockdown of MBP-1 in human prostate cancer cells delays cell cycle progression, J Biol Chem 281 (2006) 23652-7.

12. Y. Song, Q. Luo, H. Long, Z. Hu, T. Que, X. Zhang, Z. Li, G. Wang, L. Yi, Z. Liu, W. Fang,S. Qi, Alpha-enolase as a potential cancer prognostic marker promotes cell growth, migration, and invasion in glioma, Mol Cancer 13 (2014) 65.
13. G.C. Chang, K.J. Liu, C.L. Hsieh, T.S. Hu, S. Charoenfuprasert, H.K. Liu, K.T. Luh, L.H. Hsu, C.W. Wu, C.C. Ting, C.Y. Chen, K.C. Chen, T.Y. Yang, T.Y. Chou, W.H. Wang, J. Whang-Peng,N.Y. Shih, Identification of alpha-enolase as an autoantigen in lung cancer: its overexpression is associated with clinical outcomes, Clin Cancer Res 12 (2006) 5746-54.
14. L.S. Li, H. Kim, H. Rhee, S.H. Kim, D.H. Shin, K.Y. Chung, K.S. Park, Y.K. Paik,J. Chang, Proteomic analysis distinguishes basaloid carcinoma as a distinct subtype of nonsmall cell lung carcinoma, Proteomics 4 (2004) 3394-400.
15. L. Yu, J. Shen, K. Mannoor, M. Guarnera,F. Jiang, Identification of ENO1 as a potential sputum biomarker for early-stage lung cancer by shotgun proteomics, Clin Lung Cancer 15 (2014) 372-378 e1.
16. N.M. White, O. Masui, L.V. Desouza, O. Krakovska, S. Metias, A.D. Romaschin, R.J. Honey, R. Stewart, K. Pace, J. Lee, M.A. Jewett, G.A. Bjarnason, K.W. Siu,G.M. Yousef, Quantitative proteomic analysis reveals potential diagnostic markers and pathways involved in pathogenesis of renal cell carcinoma, Oncotarget 5 (2014) 506-18.
17. J. Yang, M. Zhou, R. Zhao, S. Peng, Z. Luo, X. Li, L. Cao, K. Tang, J. Ma, W. Xiong, S. Fan, D.C. Schmitt, M. Tan,G. Li, Identification of candidate biomarkers for the early detection of nasopharyngeal carcinoma by quantitative proteomic analysis, J Proteomics 109 (2014) 162-75.
18. R. Rucksaken, C. Pairojkul, P. Pinlaor, N. Khuntikeo, S. Roytrakul, C. Selmi,S. Pinlaor, Plasma autoantibodies against heat shock protein 70, enolase 1 and ribonuclease/angiogenin inhibitor 1 as potential biomarkers for cholangiocarcinoma, PloS one 9 (2014) e103259.
19. K.C. Sedoris, S.D. Thomas,D.M. Miller, c-myc promoter binding protein regulates the cellular response to an altered glucose concentration, Biochemistry 46 (2007) 8659-68.
20. E. Morgan, P. Kannan-Thulasiraman,N. Noy, Involvement of Fatty Acid Binding Protein 5 and PPARbeta/delta in Prostate Cancer Cell Growth, PPAR Res 2010 (2010)
21. S.E. O'Sullivan,M. Kaczocha, FABP5 as a novel molecular target in prostate cancer, Drug Discov Today 2020)
22. J.F. Lin, J. Xu, H.Y. Tian, X. Gao, Q.X. Chen, Q. Gu, G.J. Xu, J.D. Song,F.K. Zhao, Identification of candidate prostate cancer biomarkers in prostate needle biopsy specimens using proteomic analysis, Int J Cancer 121 (2007) 2596-605.
23. J.S. Myers, A.K. von Lersner,Q.X. Sang, Proteomic Upregulation of Fatty Acid Synthase and Fatty Acid Binding Protein 5 and Identification of Cancer- and Race-Specific Pathway Associations in Human Prostate Cancer Tissues, J Cancer 7 (2016) 1452-64.
24. K. Davalieva, I.M. Kostovska, S. Kiprijanovska, K. Markoska, K. Kubelka-Sabit, V. Filipovski, S. Stavridis, O. Stankov, S. Komina, G. Petrusevska,M. Polenakovic, Proteomics analysis of malignant and benign prostate tissue by 2D DIGE/MS reveals new insights into proteins involved in prostate cancer, Prostate 75 (2015) 1586-600.
25. E.A. Morgan, S.S. Forootan, J. Adamson, C.S. Foster, H. Fujii, M. Igarashi, C. Beesley, P.H. Smith,Y. Ke, Expression of cutaneous fatty acid-binding protein (C-FABP) in prostate cancer: potential prognostic marker and target for tumourigenicity-suppression, Int J Oncol 32 (2008) 767-75.
26. K. Fujita, H. Kume, K. Matsuzaki, A. Kawashima, T. Ujike, A. Nagahara, M. Uemura, Y. Miyagawa, T. Tomonaga,N. Nonomura, Proteomic analysis of urinary extracellular vesicles from high Gleason score prostate cancer, Sci Rep 7 (2017) 42961.
27. J. Pang, W.P. Liu, X.P. Liu, L.Y. Li, Y.Q. Fang, Q.P. Sun, S.J. Liu, M.T. Li, Z.L. Su,X. Gao, Profiling protein markers associated with lymph node metastasis in prostate cancer by DIGE-based proteomics analysis, J Proteome Res 9 (2010) 216-26.

28. D.K. Vanaja, K.V. Ballman, B.W. Morlan, J.C. Cheville, R.M. Neumann, M.M. Lieber, D.J. Tindall,C.Y. Young, PDLIM4 repression by hypermethylation as a potential biomarker for prostate cancer, Clin Cancer Res 12 (2006) 1128-36.
29. K. Nitschke, P. Erben, F. Waldbillig, A. Abdelhadi, C.A. Weis, M. Gottschalt, S. Wahby, P. Nuhn, M. Boutros, M.S. Michel, J. von Hardenberg,T.S. Worst, Clinical relevance of gene expression in localized and metastatic prostate cancer exemplified by FABP5, World J Urol 38 (2020) 637-645.
30. G. Carbonetti, T. Wilpshaar, J. Kroonen, K. Studholme, C. Converso, S. d'Oelsnitz,M. Kaczocha, FABP5 coordinates lipid signaling that promotes prostate cancer metastasis, Sci Rep 9 (2019) 18944.
31. W. Al-Jameel, X. Gou, S.S. Forootan, M.S. Al Fayi, P.S. Rudland, F.S. Forootan, J. Zhang, P.A. Cornford, S.A. Hussain,Y. Ke, Inhibitor SBFI26 suppresses the malignant progression of castration-resistant PC3-M cells by competitively binding to oncogenic FABP5, Oncotarget 8 (2017) 31041-31056.
32. W. Al-Jameel, X. Gou, X. Jin, J. Zhang, Q. Wei, J. Ai, H. Li, A. Al-Bayati, A. Platt-Higgins, A. Pettitt, P.S. Rudland,Y. Ke, Inactivated FABP5 suppresses malignant progression of prostate cancer cells by inhibiting the activation of nuclear fatty acid receptor PPARgamma, Genes Cancer 10 (2019) 80-96.
33. M. Ostergaard, H.H. Rasmussen, H.V. Nielsen, H. Vorum, T.F. Orntoft, H. Wolf,J.E. Celis, Proteome profiling of bladder squamous cell carcinomas: identification of markers that define their degree of differentiation, Cancer Res 57 (1997) 4111-7.
34. H.H. Rasmussen, T.F. Orntoft, H. Wolf,J.E. Celis, Towards a comprehensive database of proteins from the urine of patients with bladder cancer, J Urol 155 (1996) 2113-9.
35. R.S. Uma, K.N. Naresh, A.K. D'Cruz, R. Mulherkar,A.M. Borges, Metastasis of squamous cell carcinoma of the oral tongue is associated with down-regulation of epidermal fatty acid binding protein (E-FABP), Oral Oncol 43 (2007) 27-32.
36. Z. Li, W. Min, C. Huang, S. Bai, M. Tang,X. Zhao, Proteomics-based approach identified differentially expressed proteins with potential roles in endometrial carcinoma, Int J Gynecol Cancer 20 (2010) 9-15.
37. Z. Li, C. Huang, S. Bai, X. Pan, R. Zhou, Y. Wei,X. Zhao, Prognostic evaluation of epidermal fatty acid-binding protein and calcyphosine, two proteins implicated in endometrial cancer using a proteomic approach, Int J Cancer 123 (2008) 2377-83.
38. H. Li, Q. Lu, L.H. Dong, H. Xue, H.Y. Zhou,H.J. Yang, [Expression of fatty acid binding protein in human breast cancer tissues], Xi Bao Yu Fen Zi Mian Yi Xue Za Zhi 23 (2007) 312-6.
39. I.H. McKillop, C.A. Girardi,K.J. Thompson, Role of fatty acid binding proteins (FABPs) in cancer development and progression, Cell Signal 62 (2019) 109336.
40. X. Wei, X. Liu, C. Tan, L. Mo, H. Wang, X. Peng, F. Deng,L. Chen, Expression and Function of Zinc-alpha2-Glycoprotein, Neurosci Bull 35 (2019) 540-550.
41. C. Bing, Y. Bao, J. Jenkins, P. Sanders, M. Manieri, S. Cinti, M.J. Tisdale,P. Trayhurn, Zinc-alpha2-glycoprotein, a lipid mobilizing factor, is expressed in adipocytes and is up-regulated in mice with cancer cachexia, Proceedings of the National Academy of Sciences of the United States of America 101 (2004) 2500-5.
42. B. Kong, C.W. Michalski, X. Hong, N. Valkovskaya, S. Rieder, I. Abiatari, S. Streit, M. Erkan, I. Esposito, H. Friess,J. Kleeff, AZGP1 is a tumor suppressor in pancreatic cancer inducing mesenchymal-to-epithelial transdifferentiation by inhibiting TGF-beta-mediated ERK signaling, Oncogene 29 (2010) 5146-58.
43. S.D. Garbis, S.I. Tyritzis, T. Roumeliotis, P. Zerefos, E.G. Giannopoulou, A. Vlahou, S. Kossida, J. Diaz, S. Vourekas, C. Tamvakopoulos, K. Pavlakis, D. Sanoudou,C.A. Constantinides, Search for potential markers for prostate cancer diagnosis, prognosis and treatment in clinical tissue specimens using amine-specific isobaric tagging (iTRAQ) with two-dimensional liquid chromatography and tandem mass spectrometry, J Proteome Res 7 (2008) 3146-58.
44. R. Ummanni, H. Junker, U. Zimmermann, S. Venz, S. Teller, J. Giebel, C. Scharf, C. Woenckhaus, F. Dombrowski,R. Walther, Prohibitin identified by proteomic analysis of prostate biopsies distinguishes hyperplasia and cancer, Cancer Lett 266 (2008) 171-85.
45. I. Rehman, C.A. Evans, A. Glen, S.S. Cross, C.L. Eaton, J. Down, G. Pesce, J.T. Phillips, O.S. Yen, G.N. Thalmann, P.C. Wright,F.C. Hamdy, iTRAQ identification of candidate serum biomarkers associated with metastatic progression of human prostate cancer, PloS one 7 (2012) e30885.

46. J.C. Byrne, M.R. Downes, N. O'Donoghue, C. O'Keane, A. O'Neill, Y. Fan, J.M. Fitzpatrick, M. Dunn,R.W. Watson, 2D-DIGE as a strategy to identify serum markers for the progression of prostate cancer, J Proteome Res 8 (2009) 942-57.
47. I. Katafigiotis, S.I. Tyritzis, K.G. Stravodimos, C. Alamanis, K. Pavlakis, A. Vlahou, M. Makridakis, A. Katafigioti, S.D. Garbis,C.A. Constantinides, Zinc alpha2-glycoprotein as a potential novel urine biomarker for the early diagnosis of prostate cancer, BJU Int 110 (2012) E688-93.
48. G. O'Hurley, A. O'Grady, P. Smyth, J. Byrne, J.J. O'Leary, O. Sheils, R. William, G. Watson,E.W. Kay, Evaluation of Zinc-alpha-2-Glycoprotein and Proteasome Subunit beta-Type 6 Expression in Prostate Cancer Using Tissue Microarray Technology, Appl Immunohistochem Mol Morphol 18 (2010) 512-7.
49. J. Chen, J. Xi, Y. Tian, G.S. Bova,H. Zhang, Identification, prioritization, and evaluation of glycoproteins for aggressive prostate cancer using quantitative glycoproteomics and antibody-based assays on tissue specimens, Proteomics 13 (2013) 2268-77.
50. Y. Liu, J. Chen, A. Sethi, Q.K. Li, L. Chen, B. Collins, L.C. Gillet, B. Wollscheid, H. Zhang,R. Aebersold, Glycoproteomic analysis of prostate cancer tissues by SWATH mass spectrometry discovers N-acylethanolamine acid amidase and protein tyrosine kinase 7 as signatures for tumor aggressiveness, Mol Cell Proteomics 13 (2014) 1753-68.
51. S.M. Henshall, L.G. Horvath, D.I. Quinn, S.A. Eggleton, J.J. Grygiel, P.D. Stricker, A.V. Biankin, J.G. Kench,R.L. Sutherland, Zinc-alpha2-glycoprotein expression as a predictor of metastatic prostate cancer following radical prostatectomy, J Natl Cancer Inst 98 (2006) 1420-4.
52. J. Lapointe, C. Li, J.P. Higgins, M. van de Rijn, E. Bair, K. Montgomery, M. Ferrari, L. Egevad, W. Rayford, U. Bergerheim, P. Ekman, A.M. DeMarzo, R. Tibshirani, D. Botstein, P.O. Brown, J.D. Brooks,J.R. Pollack, Gene expression profiling identifies clinically relevant subtypes of prostate cancer, Proceedings of the National Academy of Sciences of the United States of America 101 (2004) 811-6.
53. P.Y. Yip, J.G. Kench, K.K. Rasiah, R.P. Benito, C.S. Lee, P.D. Stricker, S.M. Henshall, R.L. Sutherland,L.G. Horvath, Low AZGP1 expression predicts for recurrence in margin-positive, localized prostate cancer, Prostate 71 (2011) 1638-45.
54. G. Kristensen, K.D. Berg, B.G. Toft, H.V. Stroomberg, R. Nolley, J.D. Brooks, K. Brasso,M.A. Roder, Predictive value of AZGP1 following radical prostatectomy for prostate cancer: a cohort study and meta-analysis, J Clin Pathol 72 (2019) 696-704.
55. S. Irmak, D. Tilki, J. Heukeshoven, L. Oliveira-Ferrer, M. Friedrich, H. Huland,S. Ergun, Stage-dependent increase of orosomucoid and zinc-alpha2-glycoprotein in urinary bladder cancer, Proteomics 5 (2005) 4296-304.
56. J.W. Choi, H. Liu, D.H. Shin, G.I. Yu, J.S. Hwang, E.S. Kim,J.W. Yun, Proteomic and cytokine plasma biomarkers for predicting progression from colorectal adenoma to carcinoma in human patients, Proteomics 13 (2013) 2361-74.
57. A.K. Mu, B.K. Lim, O.H. Hashim,A.S. Shuib, Detection of differential levels of proteins in the urine of patients with endometrial cancer: analysis using two-dimensional gel electrophoresis and o-glycan binding lectin, Int J Mol Sci 13 (2012) 9489-9501.
58. R. Rajpal, P. Dowling, J. Meiller, C. Clarke, W.G. Murphy, R. O'Connor, M. Kell, C. Mitsiades, P. Richardson, K.C. Anderson, M. Clynes,P. O'Gorman, A novel panel of protein biomarkers for predicting response to thalidomide-based therapy in newly diagnosed multiple myeloma patients, Proteomics 11 (2011) 1391-402.
59. R.J. Skipworth, G.D. Stewart, M. Bhana, J. Christie, C.M. Sturgeon, D.C. Guttridge, A.D. Cronshaw, K.C. Fearon,J.A. Ross, Mass spectrometric detection of candidate protein biomarkers of cancer cachexia in human urine, Int J Oncol 36 (2010) 973-82.
60. A. Vidotto, T. Henrique, L.S. Raposo, J.V. Maniglia,E.H. Tajara, Salivary and serum proteomics in head and neck carcinomas: before and after surgery and radiotherapy, Cancer Biomark 8 (2010) 95-107.
61. J.D. Hayes,R.C. Strange, Glutathione S-transferase polymorphisms and their biological consequences, Pharmacology 61 (2000) 154-66.
62. L. Gate,K.D. Tew, Glutathione S-transferases as emerging therapeutic targets, Expert Opin Ther Targets 5 (2001) 477-489.
63. V. Adler,M.R. Pincus, Effector peptides from glutathione-S-transferase-pi affect the activation of jun by jun-N-terminal kinase, Ann Clin Lab Sci 34 (2004) 35-46.

64. F.N. Jiang, H.C. He, Y.Q. Zhang, D.L. Yang, J.H. Huang, Y.X. Zhu, R.J. Mo, G. Chen, S.B. Yang, Y.R. Chen, W.D. Zhong,W.L. Zhou, An integrative proteomics and interaction network-based classifier for prostate cancer diagnosis, PloS one 8 (2013) e63941.
65. D.G. Bostwick, I. Meiers,J.H. Shanks, Glutathione S-transferase: differential expression of alpha, mu, and pi isoenzymes in benign prostate, prostatic intraepithelial neoplasia, and prostatic adenocarcinoma, Hum Pathol 38 (2007) 1394-401.
66. N. Ashour, J.C. Angulo, G. Andres, R. Alelu, A. Gonzalez-Corpas, M.V. Toledo, J.M. Rodriguez-Barbero, J.I. Lopez, M. Sanchez-Chapado,S. Ropero, A DNA hypermethylation profile reveals new potential biomarkers for prostate cancer diagnosis and prognosis, Prostate 74 (2014) 1171-82.
67. J.C. Angulo, J.I. Lopez, J.F. Dorado, M. Sanchez-Chapado, B. Colas,S. Ropero, A DNA Hypermethylation Profile Independently Predicts Biochemical Recurrence Following Radical Prostatectomy, Urol Int 97 (2016) 16-25.
68. P.G. Patel, T. Wessel, A. Kawashima, J.B.A. Okello, T. Jamaspishvili, K.P. Guerard, L. Lee, A.Y. Lee, N.E. How, D. Dion, E. Scarlata, C.L. Jackson, S. Boursalie, T. Sack, R. Dunn, M. Moussa, K. Mackie, A. Ellis, E. Marra, J. Chin, K. Siddiqui, K. Hetou, L.A. Pickard, V. Arthur-Hayward, G. Bauman, S. Chevalier, F. Brimo, P.C. Boutros, D.J. Lapointe Ph, J.M.S. Bartlett, R.J. Gooding,D.M. Berman, A three-gene DNA methylation biomarker accurately classifies early stage prostate cancer, Prostate 79 (2019) 1705-1714.
69. N. Fouz, A. Amid,Y.Z. Hashim, Gene expression analysis in MCF-7 breast cancer cells treated with recombinant bromelain, Appl Biochem Biotechnol 173 (2014) 1618-39.
70. S.C. Tang, C.H. Wu, C.H. Lai, W.W. Sung, W.J. Yang, L.C. Tang, C.P. Hsu,J.L. Ko, Glutathione S-transferase mu2 suppresses cancer cell metastasis in non-small cell lung cancer, Mol Cancer Res 11 (2013) 518-29.
71. T. Simic, A. Savic-Radojevic, M. Pljesa-Ercegovac, M. Matic,J. Mimic-Oka, Glutathione S-transferases in kidney and urinary bladder tumors, Nat Rev Urol 6 (2009) 281-9.
72. J. Cui, G. Li, J. Yin, L. Li, Y. Tan, H. Wei, B. Liu, L. Deng, J. Tang, Y. Chen,L. Yi, GSTP1 and cancer: Expression, methylation, polymorphisms and signaling (Review), Int J Oncol 56 (2020) 867-878.
73. A. Latosinska, K. Davalieva, M. Makridakis, W. Mullen, J.P. Schanstra, A. Vlahou, H. Mischak,M. Frantzi, Molecular Changes in Tissue Proteome during Prostate Cancer Development: Proof-of-Principle Investigation, Diagnostics (Basel) 10 (2020)
74. Z.D. Han, Y.Q. Zhang, H.C. He, Q.S. Dai, G.Q. Qin, J.H. Chen, C. Cai, X. Fu, X.C. Bi, J.G. Zhu, D.J. Liao, X.P. Lu, Z.Y. Mo, Y.P. Zhu,W.D. Zhong, Identification of novel serological tumor markers for human prostate cancer using integrative transcriptome and proteome analysis, Med Oncol 29 (2012) 2877-88.
75. F. Martignano, G. Gurioli, S. Salvi, D. Calistri, M. Costantini, R. Gunelli, U. De Giorgi, F. Foca,V. Casadio, GSTP1 Methylation and Protein Expression in Prostate Cancer: Diagnostic Implications, Dis Markers 2016 (2016) 4358292.
76. W. Zhang, H. Jiao, X. Zhang, R. Zhao, F. Wang, W. He, H. Zong, Q. Fan,L. Wang, Correlation between the expression of DNMT1, and GSTP1 and APC, and the methylation status of GSTP1 and APC in association with their clinical significance in prostate cancer, Mol Med Rep 12 (2015) 141-6.
77. C. Moreira-Barbosa, D. Barros-Silva, P. Costa-Pinheiro, J. Torres-Ferreira, V. Constancio, R. Freitas, J. Oliveira, L. Antunes, R. Henrique,C. Jeronimo, Comparing diagnostic and prognostic performance of two-gene promoter methylation panels in tissue biopsies and urines of prostate cancer patients, Clin Epigenetics 10 (2018) 132.
78. L. Van Neste, J. Groskopf, W.E. Grizzle, G.W. Adams, M.S. DeGuenther, P.N. Kolettis, J.E. Bryant, G.P. Kearney, M.C. Kearney, W. Van Criekinge,S.M. Gaston, Epigenetic risk score improves prostate cancer risk assessment, Prostate 77 (2017) 1259-1264.
79. F. Zhao, E. Olkhov-Mitsel, S. Kamdar, R. Jeyapala, J. Garcia, R. Hurst, M.Y. Hanna, R. Mills, A.V. Tuzova, E. O'Reilly, S. Kelly, C. Cooper, D. Brewer, A.S. Perry, J. Clark, N. Fleshner,B. Bapat, A urine-based DNA methylation assay, ProCUrE, to identify clinically significant prostate cancer, Clin Epigenetics 10 (2018) 147.

80. K. Litovkin, A. Van Eynde, S. Joniau, E. Lerut, A. Laenen, T. Gevaert, O. Gevaert, M. Spahn, B. Kneitz, P. Gramme, T. Helleputte, S. Isebaert, K. Haustermans,M. Bollen, DNA Methylation-Guided Prediction of Clinical Failure in High-Risk Prostate Cancer, PloS one 10 (2015) e0130651.
81. V. Fiano, D. Zugna, C. Grasso, M. Trevisan, L. Delsedime, L. Molinaro, P. Cassoni, M. Papotti, F. Merletti, O. Akre, A. Pettersson, L. De Marco,L. Richiardi, DNA methylation in repeat negative prostate biopsies as a marker of missed prostate cancer, Clin Epigenetics 11 (2019) 152.
82. W.H. Lee, R.A. Morton, J.I. Epstein, J.D. Brooks, P.A. Campbell, G.S. Bova, W.S. Hsieh, W.B. Isaacs,W.G. Nelson, Cytidine methylation of regulatory sequences near the pi-class glutathione S-transferase gene accompanies human prostatic carcinogenesis, Proceedings of the National Academy of Sciences of the United States of America 91 (1994) 11733-7.
83. R.A. Haroun, N.I. Zakhary, M.R. Mohamed, A.M. Abdelrahman, E.I. Kandil,K.A. Shalaby, Assessment of the prognostic value of methylation status and expression levels of FHIT, GSTP1 and p16 in non-small cell lung cancer in Egyptian patients, Asian Pac J Cancer Prev 15 (2014) 4281-7.
84. T. Pongtheerat, S. Pakdeethai, W. Purisa, S. Chariyalertsak,S. Petmitr, Promoter methylation and genetic polymorphism of glutathione S-transferase P1 gene (GSTP1) in Thai breast- cancer patients, Asian Pac J Cancer Prev 12 (2011) 2731-4.
85. N. Nishida, M. Kudo, T. Nagasaka, I. Ikai,A. Goel, Characteristic patterns of altered DNA methylation predict emergence of human hepatocellular carcinoma, Hepatology 56 (2012) 994-1003.
86. R. Sacristan, C. Gonzalez, J.M. Fernandez-Gomez, F. Fresno, S. Escaf,M. Sanchez-Carbayo, Molecular classification of non-muscle-invasive bladder cancer (pTa low-grade, pT1 low-grade, and pT1 high-grade subgroups) using methylation of tumor-suppressor genes, J Mol Diagn 16 (2014) 564-572.
87. S. Ait-El-Mkadem, M. Dayem-Quere, M. Gusic, A. Chaussenot, S. Bannwarth, B. Francois, E.C. Genin, K. Fragaki, C.L.M. Volker-Touw, C. Vasnier, V. Serre, K.L.I. van Gassen, F. Lespinasse, S. Richter, G. Eisenhofer, C. Rouzier, F. Mochel, A. De Saint-Martin, M.T. Abi Warde, M.G.M. de Sain-van der Velde, J.J.M. Jans, J. Amiel, Z. Avsec, C. Mertes, T.B. Haack, T. Strom, T. Meitinger, P.E. Bonnen, R.W. Taylor, J. Gagneur, P.M. van Hasselt, A. Rotig, A. Delahodde, H. Prokisch, S.A. Fuchs,V. Paquis-Flucklinger, Mutations in MDH2, Encoding a Krebs Cycle Enzyme, Cause Early-Onset Severe Encephalopathy, Am J Hum Genet 100 (2017) 151-159.
88. A. Glen, C.S. Gan, F.C. Hamdy, C.L. Eaton, S.S. Cross, J.W. Catto, P.C. Wright,I. Rehman, iTRAQ-facilitated proteomic analysis of human prostate cancer cells identifies proteins associated with progression, J Proteome Res 7 (2008) 897-907.
89. C. Geisler, N.T. Gaisa, D. Pfister, S. Fuessel, G. Kristiansen, T. Braunschweig, S. Gostek, B. Beine, H.C. Diehl, A.M. Jackson, C.H. Borchers, A. Heidenreich, H.E. Meyer, R. Knuchel,C. Henkel, Identification and validation of potential new biomarkers for prostate cancer diagnosis and prognosis using 2D-DIGE and MS, Biomed Res Int 2015 (2015) 454256.
90. Q. Liu, C.T. Harvey, H. Geng, C. Xue, V. Chen, T.M. Beer,D.Z. Qian, Malate dehydrogenase 2 confers docetaxel resistance via regulations of JNK signaling and oxidative metabolism, Prostate 73 (2013) 1028-37.
91. C. Li, C. He, Y. Xu, H. Xu, Y. Tang, H. Chavan, S. Duan, A. Artigues, M.L. Forrest, P. Krishnamurthy, S. Han, J.M. Holzbeierlein,B. Li, Alternol eliminates excessive ATP production by disturbing Krebs cycle in prostate cancer, Prostate 79 (2019) 628-639.
92. M.Z. Islam Khan, S.Y. Tam, Z. Azam,H.K.W. Law, Proteomic profiling of metabolic proteins as potential biomarkers of radioresponsiveness for colorectal cancer, J Proteomics 262 (2022) 104600.
93. J. Clucas,F. Valderrama, ERM proteins in cancer progression, J Cell Sci 127 (2014) 267-75.
94. Y. Song, X. Ma, M. Zhang, M. Wang, G. Wang, Y. Ye,W. Xia, Ezrin Mediates Invasion and Metastasis in Tumorigenesis: A Review, Front Cell Dev Biol 8 (2020) 588801.
95. A. Valdman, X. Fang, S.T. Pang, B. Nilsson, P. Ekman,L. Egevad, Ezrin expression in prostate cancer and benign prostatic tissue, Eur Urol 48 (2005) 852-7.

96. B. Dhondt, E. Geeurickx, J. Tulkens, J. Van Deun, G. Vergauwen, L. Lippens, I. Miinalainen, P. Rappu, J. Heino, P. Ost, N. Lumen, O. De Wever,A. Hendrix, Unravelling the proteomic landscape of extracellular vesicles in prostate cancer by density-based fractionation of urine, J Extracell Vesicles 9 (2020) 1736935.
97. S.T. Pang, X. Fang, A. Valdman, G. Norstedt, A. Pousette, L. Egevad,P. Ekman, Expression of ezrin in prostatic intraepithelial neoplasia, Urology 63 (2004) 609-12.
98. Y.C. Chuan, S.T. Pang, A. Cedazo-Minguez, G. Norstedt, A. Pousette,A. Flores-Morales, Androgen induction of prostate cancer cell invasion is mediated by ezrin, J Biol Chem 281 (2006) 29938-48.
99. S. Sizemore, M. Cicek, N. Sizemore, K.P. Ng,G. Casey, Podocalyxin increases the aggressive phenotype of breast and prostate cancer cells in vitro through its interaction with ezrin, Cancer Res 67 (2007) 6183-91.
100. Y.C. Chuan, D. Iglesias-Gato, L. Fernandez-Perez, A. Cedazo-Minguez, S.T. Pang, G. Norstedt, A. Pousette,A. Flores-Morales, Ezrin mediates c-Myc actions in prostate cancer cell invasion, Oncogene 29 (2010) 1531-42.
101. R. Singh, N. Kapur, H. Mir, N. Singh, J.W. Lillard, Jr.,S. Singh, CXCR6-CXCL16 axis promotes prostate cancer by mediating cytoskeleton rearrangement via Ezrin activation and alphavbeta3 integrin clustering, Oncotarget 7 (2016) 7343-53.
102. W.A. Schulz, M. Ingenwerth, C.E. Djuidje, C. Hader, J. Rahnenfuhrer,R. Engers, Changes in cortical cytoskeletal and extracellular matrix gene expression in prostate cancer are related to oncogenic ERG deregulation, BMC Cancer 10 (2010) 505.
103. G. Wang, W. Mao,S. Zheng, MicroRNA-183 regulates Ezrin expression in lung cancer cells, FEBS Lett 582 (2008) 3663-8.
104. N. Li, J. Kong, Z. Lin, Y. Yang, T. Jin, M. Xu, J. Sun,L. Chen, Ezrin promotes breast cancer progression by modulating AKT signals, Br J Cancer 120 (2019) 703-713.
105. V. Horwitz, B. Davidson, D. Stern, C.G. Trope, T. Tavor Re'em,R. Reich, Ezrin Is Associated with Disease Progression in Ovarian Carcinoma, PloS one 11 (2016) e0162502.
106. K.P. Saavedra, P.M. Brebi,J.C. Roa, Epigenetic alterations in preneoplastic and neoplastic lesions of the cervix, Clin Epigenetics 4 (2012) 13.
107. L. Li, Y.Y. Wang, Z.S. Zhao,J. Ma, Ezrin is associated with gastric cancer progression and prognosis, Pathol Oncol Res 17 (2011) 909-15.
